# Novel Immunotherapy Options for Extranodal NK/T-Cell Lymphoma

**DOI:** 10.3389/fonc.2018.00139

**Published:** 2018-04-30

**Authors:** Boyu Hu, Yasuhiro Oki

**Affiliations:** ^1^Division of Cancer Medicine, The University of Texas MD Anderson Cancer Center, Houston, TX, United States; ^2^Division of Lymphoma and Myeloma, The University of Texas MD Anderson Cancer Center, Houston, TX, United States

**Keywords:** NK T cell lymphoma, CD30 ligand/CD30, CD38, programmed death 1, programmed death ligand 1, latent membrane protein 1, LMP2, EBV lymphoma

## Abstract

Extranodal NK/T-cell lymphoma (ENKTCL) is a highly aggressive mature NK/T-cell neoplasm marked by NK-cell phenotypic expression of CD3ε and CD56. While the disease is reported worldwide, there is a significant geographic variation with its highest incidence in East Asian countries possibly related to the frequent early childhood exposure of Epstein–Barr virus (EBV) and specific ethnic–genetical background, which contributes to the tumorigenesis. Historically, anthracycline-based chemotherapy such as CHOP (cyclophosphamide, adriamycin, vincristine, and prednisone) was used, but resulted in poor outcomes. This is due in part to intrinsic ENKTCL resistance to anthracycline caused by high expression levels of P-glycoprotein. The recent application of combined modality therapy with concurrent or sequential radiation therapy for early stage disease, along with non-anthracycline-based chemotherapy regimens consisting of drugs independent of P-glycoprotein have significantly improved clinical outcomes. Particularly, this neoplasm shows high sensitivity to l-asparaginase as NK-cells lack asparagine synthase activity. Even still, outcomes of patients with advanced stage disease or those with relapsed/recurrent disease are dismal with overall survival of generally a few months. Thus, novel therapies are needed for this population. Clinical activity of targeted antibodies along with antibody–drug conjugates, such as daratumumab (naked anti-CD38 antibody) and brentuximab vedotin (anti-CD30 antibody conjugated with auristatin E), have been reported. Further promising data have been shown with checkpoint inhibitors as high levels of programmed death-ligand 1 expression are observed in ENKTCL due to EBV-driven overexpression of the latent membrane proteins [latent membrane protein 1 (LMP1) and LMP2] with activation of the NF-κB/MAPK pathways. Initial case series with programmed death 1 inhibitors showed an overall response rate of 100% in seven relapsed patients including five with a complete response (CR). Furthermore, cellular immunotherapy with engineered cytotoxic T lymphocytes targeted against LMP1 and LMP2 have shown encouraging results with durable CRs as either maintenance therapy after initial induction chemotherapy or in the relapsed/refractory setting. In this paper, we review this exciting field of novel immunotherapy options against ENKTCL that hopefully will change the treatment paradigm in this deadly disease.

## Background

Extranodal NK/T-cell lymphoma (ENKTCL) is a locally destructive and highly aggressive mature lymphoid neoplasm with a prevalence of <1% of all non-Hodgkin’s lymphomas (NHLs) in the western world and up to 10% of NHLs in Asia and South America (1). The regional differences in prevalence is due in part to the Epstein–Barr virus (EBV) related pathogenesis of the disease and early childhood exposure to the virus (2). Furthermore, environmental and genetic factors may contribute to its etiology as recent SEER registry studies have shown higher incidence of the disease in Asian-Pacific Islanders and Hispanics as compared to non-Hispanic whites even in the United States (3, 4). Its classification and diagnosis is made by its immunophenotypic expression of CD2+, sCD3−, cytoplasmic CD3ε+, CD56+, and cytotoxic molecules, including perforin, granzyme B, and T-cell intracellular antigen 1 (1, 5, 6). EBV expression and *in situ* hybridization is imperative to the diagnosis as its presence is essential to its pathogenesis (7).

About two-thirds of the ENKTCL cases present as localized stage I and II disease mainly in the upper aerodigestive tract (UADT) (8–10). Therefore, treatment is usually a combination of chemotherapy with local radiotherapy occurring concurrently or sequentially, resulting in overall response rates of 80–90% (11–16). The 5-year progression-free survival (PFS) and overall survival (OS) rates range from 60 to 85 and 64 to 89%, respectively, which is still relatively poor for localized NHL. Over the last 20 years, the management of advanced stage ENKTCL has largely changed due to the discovery of high expression levels of P-glycoprotein on NK lymphoma cells, leading to intrinsic resistance of previously used adriamycin- and cyclophosphamide-based chemotherapy regimens (17). On the other hand, ENKTCL cells were found to lack expression of asparagine synthase and, therefore, rendered sensitive to l-asparaginase-containing chemotherapy regimens (18, 19). Even so, the complete response (CR) rates are around 50–60% with one long-term study reporting a 5-year OS of about 50% (20–23). Patients who relapse after having received l-asparaginase-containing regimens have a dismal outcome with OS of just a few months (24). Therefore, novel therapies are needed for this group of patients in the salvage setting and may even provide benefit when employed as part of a maintenance strategy in upfront therapy. The intrinsic pathogenesis of EBV-induced proliferation along with the innate expression of targetable CD markers make novel immunotherapy strategies an attractive option in l-asparaginase refractory cases. In this review, we focus on the currently available literature and case reports of immunotherapy approaches in both frontline and relapsed/refractory ENKTCL.

## Targeting CD30

Expression of CD30 has been widely reported in Hodgkin’s lymphoma (HL) and various T cell lymphomas. It functions as a member of the tumor necrosis factor receptor pathway and is not usually expressed in normal human tissue, which makes it an attractive tumor target (25). CD30 expression in ENKTCL is variable around 50–70% in three separate studies but its clinical significance remains controversial (26–28). In one 22-patient study, CD30 expression of ≥50% was associated with worse event-free survival and OS (29). In another larger 72-patient study, CD30 expression ≥5% was associated with decreased risk of relapse, increased response, and improved OS when treated with non-anthracycline-based chemotherapy (27). The largest study included 317 ENKTCL patients and out of the 91 patients who had CD30 immunohistochemistry performed on cataloged tissue, they found no association between CD30 expression and survival outcomes (26). Similarly, the most recent study of 97 patients showed 56% of specimens had CD30 expression, but there was no association with OS or PFS at CD30 cutoffs of 1, 10, or 20% (28). These variable results are due to the retrospective nature of these studies, tumor variability with varying numbers of early versus late stage patients and different cutoffs for what constitutes positive CD30 tumor expression.

Brentuximab vedotin (BV) is a CD30-targeted antibody conjugated with auristatin E that has shown high efficacy in relapsed HL and multiple T cell lymphomas (30–33). Its efficacy stems not from mechanisms of direct immune activation through the CD30 antibody but rather through the internalization of the conjugated auristatin E (MMAE) leading to direct cytotoxicity. Initial studies of a “naked” CD30 monoclonal antibody (SGN-30) showed little to no efficacy in treating CD30-positive lymphomas (34). However, responses to BV were also apparent in T cell lymphomas that had low or absent CD30 expression, suggesting that the drug may also be dispersed to the tumor microenvironment and later released into the tumor cells as a bystander effect (35, 36). Similar results were also observed in diffuse large B cell lymphoma where even two patients with ≤1% tumor CD30 expression had CRs when treated with BV (37–39). Along with direct cytotoxicity and bystander effects as mechanisms of action for BV, mouse models have shown increased immune activation after treatment with BV with enhanced T cell activation and dendritic cell priming and migration toward tumor-draining lymph nodes (40, 41). Moreover, the antitumor effects of BV were much less pronounced in immunocompromised mice. Therefore, the rational combination BV with immune-activating agents such as anti-programmed death 1 (PD1) antibodies could potentiate increased efficacy. Unfortunately, almost all patients treated with BV do eventually relapse and the means of resistance are not entirely clear as the pathogenic role of CD30 has not been fully characterized. Chen et al. showed that BV-treated HL cell lines became increasingly more resistant to MMAE (the internalized chemotherapeutic component) with the mechanism possibly being increased expression of MDR1 protein, which is a known drug exporter (42).

Although there have been no clinical trials run specifically in relapsed/refractory ENKTCL, there have been two case reports of patients achieving CR after BV therapy. One patient had non-UADT ENKTCL and was heavily pretreated who then achieved a CR after just four cycles of BV (43). The patient quickly relapsed in 3 months after discontinuing therapy due to increasing dyspnea. The other patient was a 17-year-old female also with non-UADT ENKTCL who relapsed after two cycles of an l-asparaginase-containing regimen (44). After three cycles of BV with bendamustine, she achieved a CR and was able to receive a haploidentical transplant with continued undetectable plasma EBV DNA levels posttransplant. With these encouraging results, multiple clinical trials of combining BV with both l-asparaginase and non-l-asparaginase-containing chemotherapy regimens either as sequential or combination therapy in the frontline setting have completed accrual (NCT01309789) and are being planned (NCT0324750).

Other CD30-specific therapies include engineered chimeric antigen receptor T-cells (CAR-T), which are antigen-specific T-cells with an antigen-recognizing extracellular single-chain variant fragment coupled with an activating intracellular domain that is then linked to one or more costimulatory molecules. CAR-T can be constructed toward specific targets such as CD30 and has already shown efficacy in clinical trials against various CD30-positive lymphomas. Ramos et al. treated seven HL patients and two patients with anaplastic large cell lymphoma (ALCL) with a CD28 co-stimulated anti-CD30 CAR-T with seven of these patients having previously received BV (45). Three patients achieved a CR (two HL and one ALCL) with two HL patients having stable disease. There were no reported cases of cytokine release syndrome. In another phase I trial, 18 patients (17 HL and 1 cutaneous ALCL) were treated with a 4-1BB co-stimulated anti-CD30 CAR-T construct, reporting 7 partial responses (46). Although these results are not quite as impressive as compared to their anti-CD19 CAR-T counterparts, CAR-T still remains a specific and viable treatment for relapsed/refractory ENKTCL in an area that does not have many proven options.

## Targeting CD38

CD38 is almost universally expressed within ENKTCL. In one study, investigators reported only 5% of NKTCL samples being completely negative for CD38 according to their proportion score with more than half of the samples being strongly positive (47). High CD38 expression within this disease is associated with worse PFS and OS independent of local tumor invasion. The naked anti-CD38 antibody daratumumab has high avidity toward CD38 and induces the greatest amounts of both complement-dependent cytotoxicity (CDC) and antibody-dependent cellular cytotoxicity (48). In heavily pretreated and relapsed/refractory multiple myeloma (MM) patients, daratumumab has already been approved in a variety of combinations due to high amounts of response and improvement in survival outcomes (49, 50). CD38 expression in MM has not correlated to responses to daratumumab as even patients with high proportion scores could be primary refractory, leaving the full mechanism of action of daratumumab up for further investigation (51). Response and resistance to the drug has been proposed as a combination of membrane CD38 expression, CD38/daratumumab binding, and endocytosis of the complex leading to clearance of daratumumab (51–53). More recently, upregulation of CD55 and CD59 was observed in MM patients progressing on daratumumab (54). Given the CDC mechanism of the drug, increased expression of complement inhibitory proteins, such as CD55 and CD59, suggests a method of resistance. Further supporting this, the investigators used all-trans retinoic acid to inhibit CD55/CD59 expression on relapsed MM cell lines, which then restored daratumumab CDC (54).

Activity of daratumumab in ENKTCL has been described in one case report in which a heavily pretreated patient was salvaged with the drug (55). This patient had initial stage IE disease treated with concurrent chemoradiation with a short response and widespread relapse including in the cerebrospinal fluid (CSF). She was then salvaged with an l-asparaginase-containing regimen followed by an allogeneic stem-cell transplant, but her disease relapsed 3 weeks after transplant. Daratumumab was initiated with initial rise in EBV titer levels within the first 4 weeks, but then eventual CR including clearance of her CSF at week 21. A multi-center phase II trial within multiple Asian countries is currently ongoing to assess the safety and efficacy of daratumumab within ENKTCL (NCT02927925). While most of the research on daratumumab has been in MM, ENKTCL-specific mechanisms of action and resistance will have to be investigated.

## Targeting PD1

**Figure 1:**
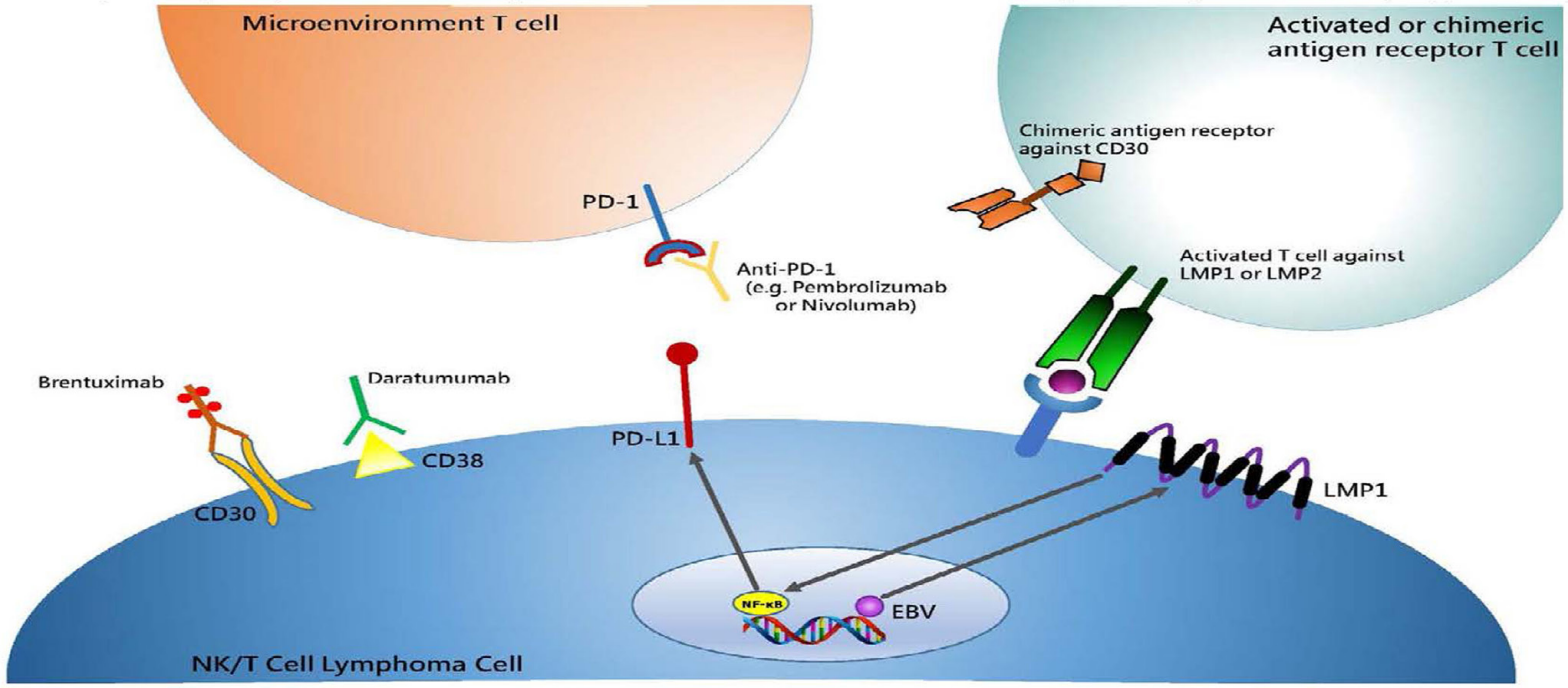
Summary of immunotherapy drugs or treatment strategies in NK/T cell lymphoma and their respective cellular membrane targets. Antibody drugs target cellular membrane proteins, which include Brentuximab/CD30 and Daratumumab/CD38. Engineered chimeric antigen T-cells are targeted toward CD30 much like Brentuximab. Anti-PD1 antibodies, such as Pembrolizumab and Nivolumab, target microenvironment T-cells that become inactivated when bound with Programmed death-ligand 1 (PD-L1) expressed on tumor cells, inducing anergy. Latent membrane protein 1 (LMP1) is a transmembrane protein produced by Epstein–Barr virus (EBV), which subsequently activates the NF-κB pathway and leads to cell proliferation and lymphomagenesis. This in turn upregulates PD-L1, which makes immune checkpoint blockade an attractive target. Furthermore, LMP1 antigen is expressed within a MHC-complex on the cell surface to which activated T cells can then recognize and extinguish. *This was an originally produced image*.

Programmed death-ligand 1 (PD-L1) is an immunomodulatory cell-surface glycoprotein mostly expressed on antigen-presenting cells (APC) as part of natural T cell anergy and downregulation (56). Multiple tumor types upregulate PD-L1 to escape immune surveillance and enhance survival. As EBV contributes to the pathogenesis of ENKTCL, expression of the immunogenic latent membrane protein 1 (LMP1) by the virus acts to enhance PD-L1 expression through upregulation of the MAPK/NF-κB pathways (Figure 1) (57). In fact, almost all of the EBV-associated lymphomas, including B cell lymphomas, were associated with high expression levels of PD-L1 (58). Reported expression of PD-L1 in ENKTCL has been variable ranging from 39 to 100% with low PD1 expression within both the tumor and infiltrating immune cells (59–62). Nodal variants of NKTCL may have higher PD-L1 expression as compared to extranodal disease (61). Correlation of PD-L1 expression to clinical characteristics has shown that increased levels were associated with lower serum LDH and IPI stage (60). Contrastingly to the association of high PD-L1 expression with traditionally classified lower risk disease, Nagato et al. reported that increased PD-L1 expression within the tumor cells was correlated with increased serum PD-L1 levels and worse OS (59). Larger studies are needed to fully correlate PD-L1 with survival as other studies have shown no association (60).

The use of anti-PD1 antibodies such as pembrolizumab and nivolumab disrupt the PD-L1/PD1 interaction and can restore the antitumor activity of activated T cells (63). Kwong et al. reported a case series of patients with previously treated ENKTCL who received pembrolizumab (64). All seven patients had been previously treated with l-asparaginase containing regimens and two patients had received allogeneic stem-cell transplants. PD-L1 expression was considered “strong” in four patients with one patient having weaker expression at 20% and the other two patients not having PD-L1 testing performed on their tumor specimens. After a median follow up of 6 months, all patients experienced an objective response with five patients achieving CR. Given the universal EBV-induced pathogenesis of ENKTCL and LMP1 directed overexpression of PD-L1, checkpoint blockade remains a very attractive immunotherapy option for this disease. Currently, multiple clinical trials are ongoing to assess the efficacy of anti-PD1 therapies in relapsed/refractory ENKTCL (NCT03107962 and NCT03021057). As suggested previously, combining anti-PD1 agents with BV could further potentiate antitumor activity.

## Targeting EBV Antigens

Further utilizing the EBV antigens present within ENKTCL, stimulated cytotoxic T lymphocytes (CTL) directed at LMP1 and LMP2 within the virus have shown efficacy in treating a multitude of EBV-derived lymphomas (65). Initial viral antigen targeting through autologous T cell activation was designed to treat viral reactivation after bone marrow transplants and was proven to be highly specific and efficacious (66, 67). Further expansion of this idea to treat EBV-associated posttransplant lymphoproliferative disorder showed sustained CRs of 68–84% (68, 69). Bollard et al. treated 52 EBV-associated lymphoma patients with a combination of LMP1/2 or LMP2-only targeted and stimulated CTLs of which 11 patients had ENKTCL (65). This study used adenoviral vector transduced and EBV-transformed APCs as stimulators for LMP-specific T cell expansion, which was later re-infused into the patients. Of the 11 patients with ENKTCL treated in this study, 6 had active disease upon CTL infusion either as primary refractory or relapsed disease. Although two of these patients had no response to the CTLs, one patient achieved a CR that later relapsed within 9 months and the remaining three had durable remissions for more than 4 years. These results are impressive in light of known data for relapsed/refractory ENKTCL patients as half had durable CRs and undetectable plasma EBV levels. The other 5 out of 11 ENKTCL patients received CTLs as consolidative therapy after initial chemoradiation or after autologous stem-cell transplantation. All five of these patients remained in CR for 2–6 years. Even as a maintenance strategy for high-risk patients, this therapy may prove highly effective as one patient who had primary refractory disease but then achieved a CR after autologous stem-cell transplant remained in CR for 2 years following CTL infusion.

While these results may be remarkable for high-risk relapsed/refractory patients, the role of CTL therapy as maintenance therapy for localized disease after first-line therapy remains to be determined. Cho et al. treated eight localized disease and two advanced disease ENKTCL with LMP1/2-directed CTLs all of whom were in CR after initial induction chemotherapy with or without radiotherapy (70). Half of the patients also had consolidative autologous stem-cell transplants. The 4-year OS and PFS were 100 and 90% with only one patient who had initial stage IVE disease relapsing after 32 months. While these results seem impressive, it is unclear if the early stage patients truly benefited from maintenance CTL infusion as historical 5-year survival rates with chemoradiation can be upwards of 90%.

## Conclusion

Extranodal NK/T-cell lymphoma remains an orphan disease with almost no phase III clinical trials to help guide therapy. Even less data are found in the relapsed/refractory cohort of patients in which most providers are using case reports and previous experience to choose treatments. With further understanding of the specific protein expression within ENKTCL, we are now able to target CD30, CD38, and PD1 as new drugs have become available (Figure 1). Combinations of these novel agents with conventional chemotherapy and each other are under investigation and may add more effective therapeutic choices. More specific to ENKTCL may be the use of EBV-antigen targeted CTLs that seem effective by themselves or as maintenance therapy for this disease. Although not yet tested in ENKTCL, CD30-targeted CAR-T may provide other T cell immunotherapy options for this disease (Table 1). Choosing one therapy over another is currently due to provider preference and patient-derived side effects from these drugs, but the overall goal would be to produce a deep response and move these relapsed/refractory patients onto an allogeneic bone marrow transplant. Future clinical trials with these novel immunotherapies will help to determine efficacy and whether to give these drugs upfront or in the salvage setting.

**Table 1:** Summary of immunotargets and drugs/therapies available against various intrinsic NK/T cell lymphoma markers or viral antigens. Best response rates are briefly summarized in the efficacy column.

Target	Drug/Therapy	Efficacy	Comment	Reference
CD30	Brentuximab	Two case reports both achieving CR		(43, 44)
CD30	Chimeric antigen receptor T cells	Mostly SD or PR with 3 patients achieving CR	Patients had either HL or ALCL, and currently remains untested in ENKTCL	(45, 46)
CD38	Daratumumab	One case report with CR		(55)
PD1	Pembrolizumab, Nivolumab	Case series with 7 patients treated. All patients achieved a response with 5 CRs		(64)
LMP1/LMP2 (EBV antigens)	Activated/stimulated T cells	6 patients had active disease with 3 patients achieving durable remissions, but 2 with no response. Maintenance strategy after first-line treatment saw durable remissions in all patients		(65, 70)

*PD1, programmed death 1; LMP, latent membrane protein; EBV, Epstein–Barr virus; CR, complete response; PR, partial response; SD, stable disease; HL, Hodgkin’s lymphoma; ALCL, anaplastic large cell lymphoma; ENKTCL, extranodal NK/T cell lymphoma*.

## Author Contributions

BH performed the literature search and wrote the manuscript for the review. He also generated the figures and tables for the manuscript. YO provided feedback, guidance and mentorship for the manuscript.

## Conflict of Interest Statement

The authors declare that the research was conducted in the absence of any commercial or financial relationships that could be construed as a potential conflict of interest.
